# Non-Hodgkin Lymphoma Presenting With Pulmonary Hyalinizing Granuloma: A Rare Clinical Entity

**DOI:** 10.7759/cureus.42907

**Published:** 2023-08-03

**Authors:** Nina Trepić, Marko Nemet, Ivan Ergelašev, Sanja Ergelašev, Dejan C Vuckovic

**Affiliations:** 1 Internal Medicine, Faculty of Medicine, University of Novi Sad, Novi Sad, SRB; 2 Thoracic Surgery, Institute for Pulmonary Diseases of Vojvodina, Sremska Kamenica, SRB; 3 Surgery, Faculty of Medicine, University of Novi Sad, Novi Sad, SRB; 4 Cardiology, Institute for Cardiovascular Diseases of Vojvodina, Sremska Kamenica, SRB; 5 Pathology, Institute for Pulmonary Diseases of Vojvodina, Sremska Kamenica, SRB; 6 Pathology, Faculty of Medicine, University of Novi Sad, Novi Sad, SRB

**Keywords:** lung resection, pet/ct, video-assisted thoracoscopy, non-hodgkin lymphoma, multiple lung nodules, pulmonary hyalinizing granuloma

## Abstract

Pulmonary hyalinizing granuloma (PHG) is an unusual benign pulmonary disease with nonspecific symptoms and slow progression, characterized by solitary or multiple fibrosing nodules. A definitive diagnosis of PHG usually requires a wide excisional biopsy. Associations of PHG with lymphoproliferative disorders, such as Castleman's disease and lymphoma, have been described. PHG is considered a paraneoplastic manifestation of those diseases. Treatment in most cases comprises therapy of the underlying condition with or without the use of empirical corticosteroid therapy. We report a case of a 57-year-old Caucasian female, who presented initially with dyspnea, fatigue, dry mouth, difficulty swallowing, night sweats, weight loss, and recurrent sinusitis. A physical examination revealed hepatosplenomegaly with generalized lymphadenomegaly. Chest computed tomography showed bilateral diffuse nodular changes about 10 mm in diameter in the lung parenchyma. A needle biopsy of a lymph node confirmed the diagnosis of non-Hodgkin lymphoma and chemotherapy was started. Since the parenchymal lung lesions progressed and no definite diagnosis could be made on the basis of transbronchial biopsy, a right-sided video-assisted thoracoscopy with atypical resection of the abnormalities was performed. The findings were consistent with a diagnosis of pulmonary hyalinizing granuloma. Due to preserved pulmonary function, there was no indication for starting corticosteroid therapy. The purpose of this case report is to draw attention to the possibility of pulmonary hyalinizing granuloma as a differential diagnosis when multiple nodular lesions are observed in the lungs. Although PHG is a rare entity, due to its frequent association with underlying diseases and nonspecific presentation, a careful investigation should be performed. For a definite diagnosis, a surgical biopsy is required.

## Introduction

Pulmonary hyalinizing granuloma (PHG) is an unusual benign pulmonary disease with nonspecific symptoms and a slow progression. It is characterized by the presence of solitary or, more commonly, multiple fibrosing nodules. Histopathological analyses reveal nodules composed of central whorled deposits of eosinophilic collagen hyaline lamellae. These nodules are surrounded by a collection of plasma cells, lymphocytes, and histiocytes. In patients with PHG, imaging studies show well-defined nodules that can be single or multiple, unilateral or bilateral. The nodules are randomly distributed, which can be confused with more frequent pulmonary disorders, such as neoplastic and granulomatous conditions [1-3]. Positron emission tomography/CT (PET/CT) can help differentiate PHG from malignant tumors, by showing non-avid lesions. However, a large excisional biopsy is frequently required to obtain a conclusive diagnosis of PHG [4]. Although the precise origin of PHG remains unknown, it is frequently linked to infectious, autoimmune, and tumoral diseases. This points to an aberrant immune response as a possible explanation for its emergence. The majority of these cases have been linked to multicentric Castleman disease [5,6], while others have been observed in conjunction with diffuse lymphocytic lymphoma of the abdomen [7] or pulmonary small lymphocytic lymphoma [8], and are considered a paraneoplastic manifestation of these conditions. In most cases, treatment involves addressing the underlying malignancy or other condition, with or without the administration of empirical corticosteroid therapy [9,10].

In this report, we present a case that is among the few documented instances of pulmonary hyalinizing granuloma associated with a lymphoproliferative disorder, a confirmed case of non-Hodgkin lymphoma. The patient was treated at the Institute for Pulmonary Diseases in Vojvodina, Republic of Serbia. Our aim with this case report is to draw attention to the possibility of pulmonary hyalinizing granuloma as a differential diagnosis for multiple nodular lung lesions. Although a rare entity, because of its frequent association with underlying diseases and nonspecific presentation, a careful investigation is warranted. Additionally, we discuss our diagnostic approach, the possible role of advanced imaging techniques, and the need for pathological confirmation.

## Case presentation

A 57-year-old Caucasian female initially sought medical attention at a local hospital due to a variety of symptoms. She had been experiencing dyspnea, fatigue, dry mouth, difficulty swallowing, and night sweats for the past four months. In addition, she reported a weight loss of 4-5 kg in the last two months and recurrent sinusitis. The patient had a smoking history of 40 pack-years but did not have any other known comorbidities or specific family history. During the physical examination, hepatosplenomegaly with generalized lymphadenomegaly was noted.

A chest CT scan revealed bilateral diffuse nodular changes approximately 10 mm in diameter across all lung lobes, accompanied by bilateral pleural effusion. Some of these nodules exhibited cavitations. The CT scan additionally identified conglomerates of enlarged lymph nodes located retro- and intraperitoneally. Following this, a needle biopsy was conducted on the enlarged lymph node in the left-sided neck region, confirming a diagnosis of non-Hodgkin lymphoma grade 3a, clinical stage (CS) IVB. Subsequently, the patient commenced R-CHOP chemotherapy (rituximab, cyclophosphamide, doxorubicin hydrochloride, vincristine sulfate, and prednisone) and achieved a partial response after three cycles (two months). The chemotherapy regimen was continued for a total of eight cycles (six months), after which a maintenance regimen of six cycles (11 months) was followed with rituximab. This resulted in the resolution of lymphadenomegaly, hepatosplenomegaly, and pleural effusion. The patient exhibited clinical, functional, and radiological improvement.

**Figure 1:**
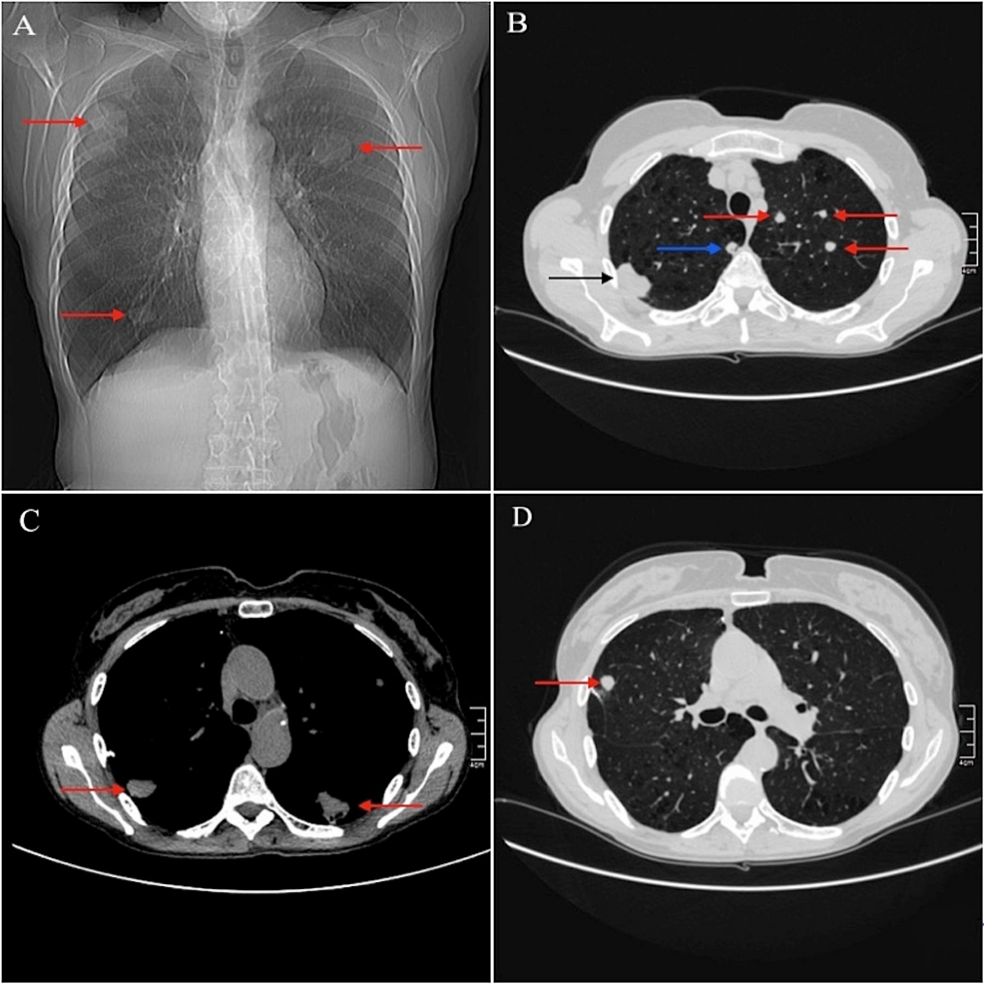
Chest CT showing multiple pulmonary nodules (A) A chest CT scout image showing multiple bilateral nodules within the lung parenchyma, indicated by red arrows; (B, D) CT images in lung window settings. A prominent right-sided lesion measuring 25 x 50 x 50 mm, located in the S2 pulmonary segment, is highlighted by a black arrow. A small right-sided nodule in the S2 pulmonary segment, in contact with the mediastinal pleura, is indicated by a blue arrow. Additional smaller bilateral nodules are depicted by red arrows. (C) A CT image in mediastinal (soft tissue) window setting. Two nodules in direct contact with the parietal pleura are marked by red arrows. The left-sided lesion exhibits partial cavitation.

A follow-up CT scan done two months after the last maintenance dose revealed progression in nodule size and the presence of several subpleural hyperdense nodules in the lung parenchyma (Figure 1). The most prominent lesions, measuring 25 x 50 x 50 mm, were observed in S2 and some were in contact with the mediastinal pleura. A transbronchial biopsy taken from the right upper lobe did not detect any tumor presence. Since the biopsies did not provide a definitive diagnosis and the changes observed on the CT scan were too small and difficult to access for further invasive pulmonary diagnosis, a thoracotomy with anatomical resection of the adjacent lobe became necessary. Before the surgical procedure, a PET/CT scan was performed to assess the metabolic activity of the nodules. The results revealed that the nodules were non-^18^F-fluorodeoxyglucose (FDG) avid.

**Figure 2:**
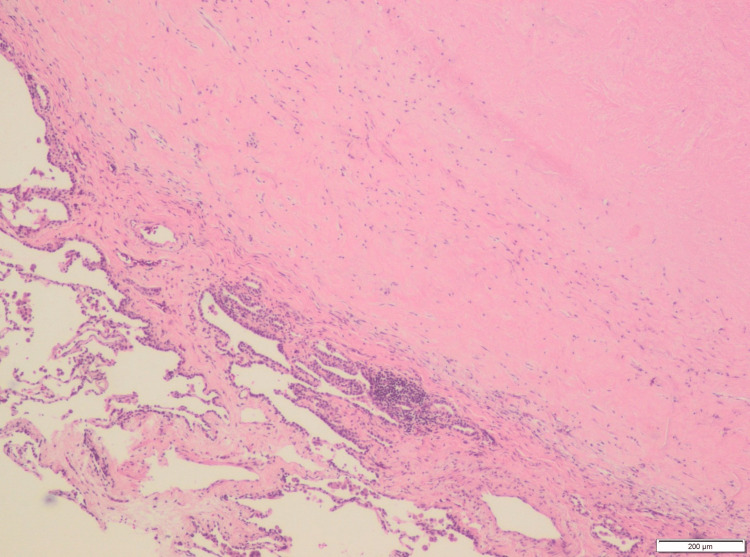
A microscopic image of a distinct nodular lesion from the lung specimen Acellular eosinophilic hyalinized collagen bundles arranged in whorls are observed under a microscopic examination (hematoxylin-eosin staining, x40).

The preferred surgical procedure was a right-sided video-assisted thoracoscopy (VATS) involving atypical resection of two abnormalities in the upper lobe and one abnormality in the lower lobe on the right side. These resected specimens were subjected to a histopathological examination, which revealed the presence of concentric whorled deposits of coarse collagen surrounded by infiltrates of lymphocytes and plasma cells (Figure 2). No necrosis, tumor elements, or granulomatous lesions were observed in the sample. These findings were consistent with the diagnosis of pulmonary hyalinizing granuloma.

The patient was referred to an oncologist to discuss the initiation of corticosteroid therapy. At the time, pulmonary function tests did not show any significant abnormalities. The forced expiratory volume in one second (FEV1) was measured at 2.47 L, which was 99% of the predicted value. The forced vital capacity (FVC) was 3.66 L, equivalent to 120% of the predicted value. The FEV1/FVC ratio was within the normal range, indicating preserved lung function. However, the carbon monoxide transfer factor (TLCO) showed a slight decrease, measuring 49%. Considering the mostly preserved pulmonary function and absence of indications, corticosteroid therapy was not initiated. The patient remained in a good overall condition without respiratory or other complaints, and is being regularly monitored by her pulmonologist.

## Discussion

In 1977, Engelman et al. defined pulmonary hyalinizing granuloma as a unique entity, a rare benign condition that is characterized by single or numerous lung nodules simulating metastatic lung disease [11]. Mild and nonspecific clinical symptoms are common in patients with pulmonary hyalinizing granuloma. In some cases, patients may be asymptomatic, and the nodules are detected incidentally during routine screening examinations. Common symptoms associated with PHG include cough, fever, fatigue, shortness of breath, pleuritic chest pain, sinusitis, and pharyngitis [12,13].

The nodules identified on CT scans in cases of PHG may exhibit different characteristics. They can be either calcified or non-calcified. In the case of calcified masses, they are often multiple and distributed bilaterally. Additionally, although rare, cavitations within the nodules can also occur. The size of the nodules can vary, ranging from a few millimeters to as large as 15 cm in diameter, with an average size of 2 cm [8].

Primary or metastatic malignancy, infection, amyloidosis, rheumatoid nodules, Wegener's granulomatosis, lymphomatoid granulomatosis, and plasma cell granuloma are all possible differential diagnoses for PHG. To differentiate PHG from these other conditions, ^18^F-FDG-PET/CT can be utilized to identify increased metabolic activity in the lesions. However, only a histopathological examination can provide a reliable diagnosis of PHG [2]. In the case described, despite the PET/CT scan revealing non-FDG avid nodules, VATS was performed to obtain a histopathological diagnosis.

PHG generally has a good prognosis, with lesions normally remaining stable for a long time [14]. Single lesions tend to have a better prognosis and can even be surgically removed for cure [15]. The progression of multiple lesions, on the other hand, may result in reduced pulmonary function and a poorer prognosis. Corticosteroids are useful in providing radiological and symptomatic improvement in most cases of PHG [14].

In the context of PHG cases associated with lymphoproliferative disorders, chemotherapy targeting the underlying disease, including corticosteroids, has been reportedly administered. This treatment approach resulted in a reduction in the dimensions of lung nodules in two out of three published cases [6-8]. In one case showing improvement, prednisolone was given at a daily dose of 25 mg [6]. In our patient, although therapy with R-CHOP was completed, the lesions continued to progress. It is worth considering that the short duration of the administered corticosteroid therapy (only four days after each cycle) may have been insufficient to halt the progression.

## Conclusions

The objective of this case report is to raise awareness about the possibility of PHG as a differential diagnosis when multiple nodular lesions are detected in the lungs. PHG is a rare condition, and due to its frequent association with underlying diseases, a thorough investigation is warranted. Given its nonspecific presentation, PHG can be easily overlooked or misdiagnosed. It is crucial to emphasize that the absence of FDG uptake in the lesions should not rule out the need for histopathological analysis. To establish an accurate diagnosis, surgical biopsy, considering the potential differential diagnoses, is essential.
